# Cholinergic input origins and current response properties in rat interstitial nucleus of Cajal neurons

**DOI:** 10.3389/fncel.2026.1925102

**Published:** 2026-10-23

**Authors:** Taketoshi Sugimura, Yasuhiko Saito

**Affiliations:** Department of Neurophysiology, Nara Medical University, Kashihara, Japan

**Keywords:** gaze holding, medial vestibular nucleus, muscarinic receptor, neural integrator, nicotinic receptor, prepositus hypoglossi nucleus, whole-cell recording

## Abstract

The interstitial nucleus of Cajal (INC) and the prepositus hypoglossi nucleus (PHN) function as the neural integrators for vertical and horizontal gaze holding, respectively. Previous studies have suggested that cholinergic inputs from the mesopontine tegmentum modulate the function of the horizontal integrator; however, the modulation properties and origins of cholinergic inputs in the vertical integrator remain unclear. In this study, we investigated the cholinergic responses of INC neurons and the anatomical origin of the cholinergic inputs in rats. Whole-cell recordings in brainstem slices revealed that local application of acetylcholine (ACh) induced inward currents in INC neurons mediated by both nicotinic and muscarinic ACh receptors (nAChRs and mAChRs). Although current responses were predominantly mediated by nAChRs in PHN neurons, the participation of nAChRs and mAChRs differed depending on the INC neuron type. Retrograde tracing using an adeno-associated virus (AAV) in choline acetyltransferase (ChAT)-tdTomato transgenic rats demonstrated that the primary origin of cholinergic inputs to the INC was the caudal magnocellular region of the medial vestibular nucleus (MVeMC). Furthermore, the primary cholinergic inputs to the PHN originated from both the caudal MVeMC and the caudal PHN itself. These results indicate that the vertical neural integrator shares a common cholinergic source in the caudal MVN with the horizontal integrator, although the relative contributions of nAChRs and mAChRs are not necessarily shared between INC and PHN neurons. These findings suggest a potential role for cholinergic modulation in both vertical and horizontal gaze holding.

## Introduction

Signals for gaze holding are generated primarily by oculomotor neural integrators that convert transient burst signals that are proportional to eye or head velocity into sustained signals that are proportional to eye position (Skavenski and Robinson, 1973; Robinson, 1975, 1989; Fukushima, 1987, 1991; Fukushima et al., 1992; Fukushima and Kaneko, 1995; Moschovakis, 1997; Leigh and Zee, 2015; Shadmehr, 2017). The prepositus hypoglossi nucleus (PHN) and the interstitial nucleus of Cajal (INC) are included in neural integrators for horizontal and vertical gaze holding, respectively (Fukushima, 1987, 1991; Fukushima et al., 1992; Fukushima and Kaneko, 1995; Moschovakis, 1997; Leigh and Zee, 2015; Sanchez and Rowe, 2018).

Although the neural basis and mechanism of neural integrators have not been fully clarified, previous studies of the PHN suggest that cholinergic inputs participate in the function of the integrator (Navarro-López et al., 2004, 2005; Delgado-García et al., 2006). Previous anatomical studies have indicated connections between the PHN and the mesopontine tegmentum (Higo et al., 1990; Iwasaki et al., 1999). This region, particularly the pedunculopontine tegmental nucleus (PPTg) and the laterodorsal tegmental nucleus (LDTg), contains prominent populations of cholinergic neurons. Together with the evidence that the PHN receives cholinergic innervation (McCrea and Horn, 2006), it has been suggested that the mesopontine tegmentum is the primary source of cholinergic inputs to the PHN (Navarro-López et al., 2004; Delgado-García et al., 2006). Pharmacological analyses of rat brainstem slice preparations using agonists and antagonists of muscarinic acetylcholine (ACh) receptors (mAChRs) revealed that the sustained activity necessary for gaze holding in PHN neurons was mediated primarily by mAChRs (Navarro-López et al., 2004). Furthermore, *in vivo* experiments in cats demonstrated that the injection of antagonists of mAChRs into the PHN induced a centripetal drift from the eye position reached after saccade, resulting in an inability to maintain the eye position (Navarro-López et al., 2004). Our previous studies in rat brainstem slices demonstrated that the local application of ACh to PHN neurons induced inward currents that were mediated not only via mAChRs but also via nicotinic acetylcholine receptors (nAChRs) (Zhang et al., 2014, 2016). Pharmacological analyses revealed that the amplitude and charge transfer of inward currents mediated by nAChRs were greater than those mediated by mAChRs, suggesting that nAChRs also play significant roles in integrator networks, including the PHN (Zhang et al., 2014, 2016, 2018).

In contrast to the findings of cholinergic modulation in the PHN, cholinergic projections to the INC from the PPTg and LDTg have not been reported (Hallanger and Wainer, 1988). Nevertheless, in our previous study using choline acetyltransferase (ChAT)-tdTomato transgenic rats, which specifically express the fluorescent protein tdTomato in cholinergic neurons, tdTomato-expressing axonal fibers were observed in the INC (Saito et al., 2017). This observation implies that INC neurons are also modulated by cholinergic transmission; however, there has been no report of cholinergic effects on INC neurons. Therefore, we asked the following three questions: (1) Do INC neurons exhibit current responses to ACh? (2) Are these cholinergic responses also primarily mediated via nAChRs, as in the PHN, or do they exhibit different receptor dependencies depending on cell type? (3) What are the primary origins of cholinergic inputs to the INC, and how do they differ from those to the PHN? To answer these questions, we investigated the current responses of INC neurons to the local application of ACh using whole-cell recordings in rat slice preparations and determined the primary brain regions that send cholinergic inputs to the INC and the PHN by retrograde tracing using an adeno-associated virus (AAV) vector in ChAT-tdTomato transgenic rats.

## Materials and methods

All experimental procedures were approved by the Animal Care Committee of Nara Medical University, and the experiments were carried out in accordance with the guidelines outlined by the U.S. National Institutes of Health regarding the care and use of animals for experimental research (Percie du Sert et al., 2020). Every effort was made to minimize suffering and the number of animals used in these experiments. Although the analyses of INC neurons were performed using the data obtained in this study, the analyses of PHN neurons were performed using the data obtained in our previous studies (Zhang et al., 2016).

### Animals

Electrophysiological data were obtained from 8 vesicular GABA transporter (VGAT)-Venus transgenic rats (Uematsu et al., 2008; Shino et al., 2011), in which inhibitory neurons express the fluorescent protein Venus. Anatomical data were obtained from 6 ChAT-tdTomato transgenic rats. The transgenic rats were identified by PCR genotyping using previously described primers (Shino et al., 2011; Zhang et al., 2014).

### Slice preparation and whole-cell recording

The procedures for slice preparation and whole-cell patch clamp recordings were similar to those previously described (Zhang et al., 2014, 2016, 2018). Briefly, transgenic rats [18 to 21 postnatal days (PNDs)] were deeply anesthetized with isoflurane and decapitated. The brain was quickly removed from the skull of each rat and placed in an oxygenated ice-cold sucrose solution containing (in mM) 234 sucrose, 2.5 KCl, 1.25 NaH_2_PO_4_, 10 MgSO_4_, 0.5 CaCl_2_, 26 NaHCO_3_, and 11 glucose. Frontal brainstem slices (250 μm thick) including the INC were cut in the sucrose solution using a microslicer (Pro 7, Dosaka EM, Kyoto, Japan). The slices were recovered in an interface-type chamber perfused with an extracellular solution containing (in mM) 125 NaCl, 2.5 KCl, 2 CaCl_2_, 1 MgCl_2_, 1.25 NaH_2_PO_4_, 26 NaHCO_3,_ and 25 glucose and aerated with 95% O_2_ and 5% CO_2_ (pH 7.4) at 33 °C for 1 h. After recovery, the slices were incubated in aerated extracellular solution at room temperature. For recordings, each slice was transferred to a submerged recording chamber on an upright microscope (Leica DM LFS, Leica Microsystems, Wetzlar, Germany) and continuously perfused with the extracellular solution at a rate of 3 ml/min. The bath temperature was maintained at 30–32 °C using an in-line heater (SH-27A, Warner Instruments, Hamden, CT). After the neurons were identified using fluorescence optics, whole-cell recordings were performed via Nomarski microscopy using an EPC-8 patch clamp amplifier (HEKA, Darmstadt, Germany). Patch pipettes were prepared from borosilicate glass capillaries and filled with an internal solution containing (in mM) 120 K-methyl sulfate, 10 KCl, 0.2 EGTA, 2 MgATP, 0.3 NaGTP, 10 HEPES, 10 Na_2_-phosphocreatine, and 0.1 spermine, adjusted to pH 7.3 with KOH. The osmolarity of the internal solution ranged from 280 to 290 mOsm/l, and the resistance of the patch electrodes ranged from 4 to 8 MΩ in the bath solution. As in our previous studies (Zhang et al., 2014, 2016, 2018), 1 mM ACh was applied to recorded neurons, which were held at −75 mV, at 30-s intervals via pressurized air. The series resistance during the recordings was monitored by applying a short voltage pulse (−10 mV, 100 ms) prior to the ACh application. Voltage and current signals were low-pass filtered at 3 kHz and 1 kHz, respectively, and their signals were digitized at 10 kHz and 5 kHz, respectively. The data were acquired using a pClamp9 system (Molecular Devices, Foster City, CA). The calculated liquid junction potential of −5 mV was corrected. Neurons that displayed membrane potentials below −50 mV immediately after patch membrane rupture and action potential peaks higher than 0 mV were used for further analyses. Acetylcholine chloride, mecamylamine, and atropine sulfate were purchased from Sigma-Aldrich Japan (Tokyo). Other drugs were purchased from FUIJIFILM Wako Pure Chemical Inst. (Osaka, Japan). Atropine and mecamylamine were added to the extracellular solution and bath-applied.

### AAV injection and histological procedures

The procedures for AAV injection and histological analyses were similar to those previously described (Sugimura and Saito, 2021; Sugimura et al., 2024). Briefly, under inhalation anesthesia with 2–3% isoflurane, male ChAT-tdTomato rats (10–12 weeks old) were placed in a stereotaxic apparatus (Paxinos and Watson, 2018). Using a dental drill, a small hole was drilled in the cranium over the INC or the prepositus hypoglossi nucleus (PHN). A retrograde AAV vector, AAVrg-hSyn-EGFP (Addgene: 50465-AAVrg; titer ≥ 1 × 10^13^ vg/ml), was injected unilaterally into the INC or the PHN. Because the PHN and MVN are located adjacent to each other, viral spread from the PHN injection unavoidably extended into the adjacent MVN. The injection was performed using a glass micropipette (tip diameter: 50–60 μm) connected to a Hamilton syringe with polyethylene tubing and filled with oil. For the injection, the tip of the micropipette was positioned 0.8 mm lateral to the midline, 3.0 mm anterior to the lambda, at a depth of 6.0 mm for the INC; and 0.8 mm lateral to the midline, 3.4 mm posterior to the lambda, at a depth of 6.5 mm for the PHN. A total of 0.6–0.8 μL of the AAV vector was injected by applying pressure for > 5 min. After the injection, the pipette was held in position for 5 min before withdrawal to prevent backflow. Following incision suturing, an antibiotic (gentamicin ointment; MSD K.K., Tokyo, Japan) was applied to the wound, and a nonsteroidal anti-inflammatory drug (flunixin meglumine, 1.5 mg/kg; Sigma-Aldrich) was administered subcutaneously. Three weeks after AAV injection, the rats were anesthetized via isoflurane inhalation followed by an intraperitoneal injection of a mixture of medetomidine (0.15 mg/kg), midazolam (2.0 mg/kg), and butorphanol (2.5 mg/kg). The rats were then transcardially perfused with 0.01 M phosphate-buffered saline (PBS), pH 7.4, followed by 4% paraformaldehyde in 0.1 M phosphate buffer (PB). The brains were removed and postfixed in the same fixative at 4 °C for 1 d. Frontal brainstem sections (60 μm thick) were cut using a microslicer (Pro 7, Dosaka EM, Kyoto, Japan). Every third section (at 180-μm intervals) from bregma −5.04 to −12.84 mm was mounted on MAS-coated slides (Matsunami Glass, Kishiwada, Japan) with antifade medium (Fluoromount; DBS, Pleasanton, CA). Images of the brain sections were captured under a fluorescence microscope (BZ-X710, Keyence, Osaka, Japan). Nuclear boundaries were determined by comparing fluorescence and bright-field images with corresponding anatomical landmarks in the rat brain atlas (Paxinos and Watson, 2018). As described previously (Zhang et al., 2014; Sugimura et al., 2025), cholinergic cranial motor nuclei, such as the oculomotor (3N), trochlear (4N), abducens (6N), facial (7N), vagal motor (10N), and hypoglossal (12N) nuclei, natively expressed intense tdTomato fluorescence in ChAT-tdTomato rats. These prominent cholinergic structures served as reliable landmarks for defining nuclear boundaries and identifying the anatomical locations of both tdTomato- and GFP-labeled neurons.

### Data analysis

Off-line analyses of electrophysiological data were performed using Axograph software (RRID: SCR_014284). Data were used for analysis only if the change in the series resistance during the recording was within 10% of the initial value. Three current traces that were recorded from a neuron were used to measure the current amplitude and charge transfer. All values are reported as the mean ± SD. In the electrophysiological experiments, n refers to the number of neurons analyzed, whereas in the histological experiments, n refers to the number of rats analyzed. Data normality was determined using the Shapiro-Wilk test. For paired data, statistical analysis was performed using paired Student’s t tests. For unpaired data, Student’s t tests (normally distributed data) and the Mann-Whitney test (data that did not follow a normal distribution) were used. Statistical significance was defined at the level of *p* < 0.05. These analyses were performed using StatView software (ver. 5.0; Hulinks, Tokyo, Japan) and JMP software (ver. 6.0.2). Fluorescence images of the brain sections were processed and analyzed using Fiji (Schindelin et al., 2012). The numbers of double-labeled neurons were manually counted with the aid of the cell counter plugin of Fiji. The detection threshold for fluorescently labeled neurons was arbitrarily defined. To quantify EGFP/tdTomato double-labeled neurons, three mounted sections at 180-μm intervals around the peak of double-labeled neurons were selected for the MVN and the PHN. The numbers of double-labeled neurons on the ipsilateral and contralateral sides were counted separately. Neurons located within the injection sites were excluded from the cell counts.

## Results

### Current responses to acetylcholine in INC neurons

To examine ACh-induced current responses in INC neurons, whole-cell patch-clamp recordings were performed in brainstem slices prepared from VGAT-Venus transgenic rats. All INC neurons tested (*n* = 38) showed inward currents in response to the local application of ACh. The current response of an INC neuron is shown in Figure 1A. Overall, the currents in INC neurons appeared to be smaller than those in PHN neurons. Indeed, when the amplitude of the current and the charge transfer of the ACh-induced currents, which were normalized by the membrane capacitance, were compared between neurons in the INC and the previously recorded PHN (*n* = 45; reanalyzed from Zhang et al., 2016; see below for details on rat strains and cell-type identification) (Figure 1B), both were smaller in INC neurons (1.44 ± 1.64 pA/pF, 0.58 ± 0.52 pC/pF) than in PHN neurons (4.86 ± 3.14 pA/pF, *p* < 0.0001, 1.29 ± 0.91 pC/pF, *p* < 0.0001, *n* = 45). These previously recorded PHN neurons included cholinergic neurons identified by tdTomato fluorescence (*n* = 16) in addition to putative glutamatergic neurons (Venus-/tdTomato-double-negative [D-], *n* = 15) and inhibitory neurons (Venus-positive [V+], *n* = 14) recorded from VGAT-Venus × ChAT-tdTomato double-transgenic rats. In contrast, because cholinergic neurons are absent in the INC (Saito et al., 2017), the INC recordings were obtained from VGAT-Venus single-transgenic rats (rather than double-transgenic rats) and included glutamatergic (Venus-negative [V-], *n* = 18) and inhibitory (Venus-positive [V+], *n* = 20) neurons. Even when cholinergic neurons in the PHN were excluded from the comparison, both current amplitudes and charge transfers remained significantly smaller in INC neurons than in PHN neurons (amplitude: *p* < 0.0001; charge transfer: *p* = 0.0011). We further compared glutamatergic and inhibitory neurons between the INC and the PHN. The amplitude and charge transfer of glutamatergic neurons in the INC (0.94 ± 0.30 pA/pF, 0.42 ± 0.09 pC/pF) were significantly smaller than those of the neurons in the PHN (5.29 ± 2.64 pA/pF, *p* < 0.0001, 1.50 ± 0.96 pC/pF, *p* < 0.0001) (Figure 1C). Although the amplitude of inhibitory neurons in the INC (1.90 ± 2.11 pA/pF) was significantly smaller than that of the neurons in the PHN (3.96 ± 2.39 pA/pF, *p* = 0.0015), the charge was not significantly different between the neurons in the INC (0.73 ± 0.62 pC/pF) and those in the PHN (0.96 ± 0.75 pC/pF, *p* = 0.33) (Figure 1D). To confirm that our previous recording conditions were consistent with those of the present study, we recorded an additional dataset of PHN neurons using our current setup in offspring derived from crossing VGAT-Venus and ChAT-tdTomato transgenic rats (*n* = 14 for D- and *n* = 14 for V+ neurons from 2 rats). With respect to glutamatergic (D-) neurons, the new dataset did not significantly differ from the previously published dataset (Zhang et al., 2016) in response amplitude (4.68 ± 2.11 pA/pF vs. previous: 5.29 ± 2.64 pA/pF; *p* = 0.5045) or charge transfer (1.41 ± 0.76 pC/pF vs. previous: 1.50 ± 0.96 pC/pF; *p* = 0.7688). Similarly, for inhibitory (V+) neurons, no significant differences were observed in amplitude (3.88 ± 2.90 pA/pF vs. previous: 3.96 ± 2.39 pA/pF; *p* = 0.5325) or charge transfer (1.13 ± 0.70 pC/pF vs. previous: 0.96 ± 0.75 pC/pF; *p* = 0.5145). Thus, there were no significant differences between the previous and new datasets across both cell types, thereby validating the direct comparison between the INC neurons analyzed in this study and the previously recorded PHN neurons.

**FIGURE 1:**
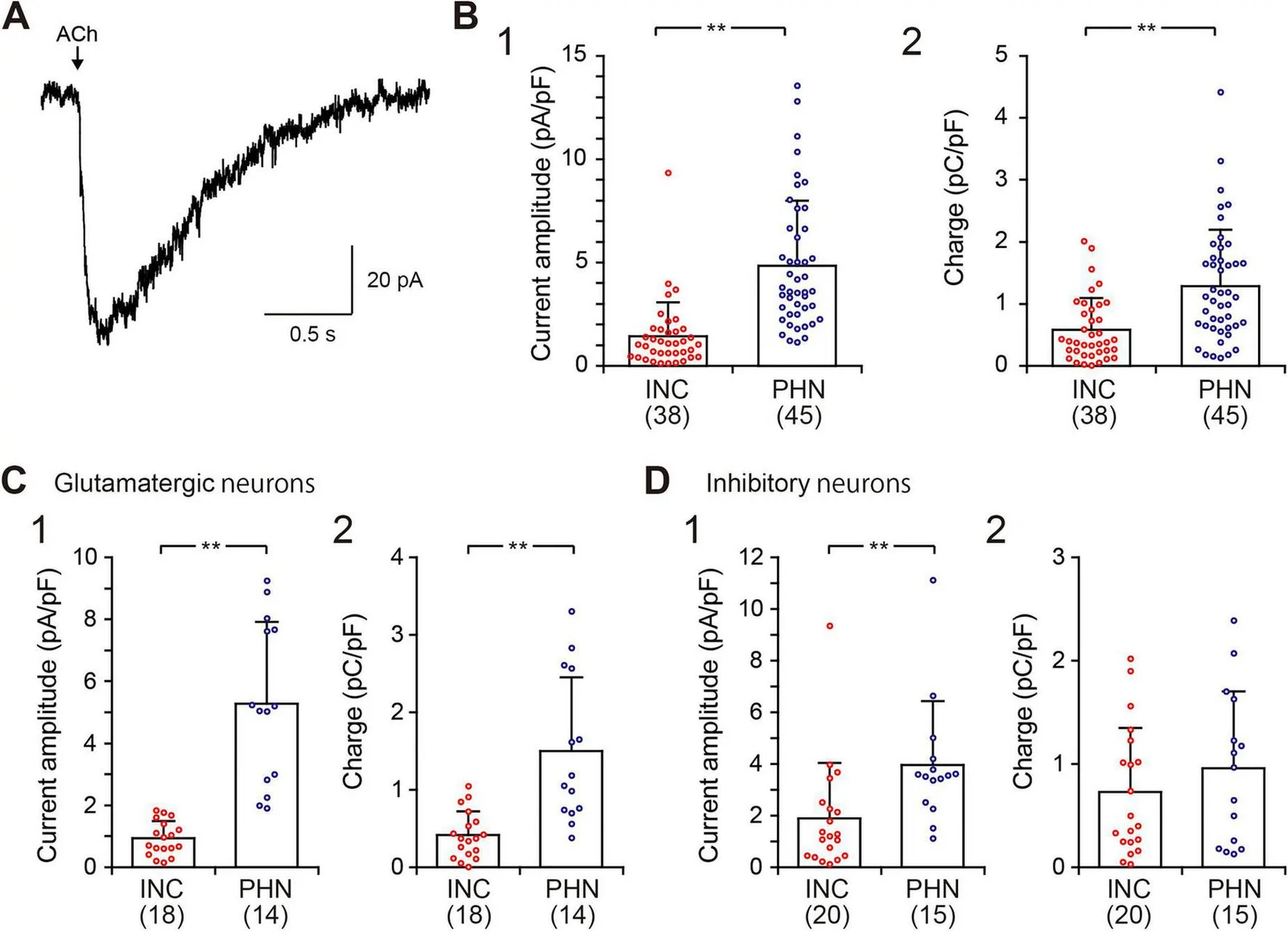
Acetylcholine-induced current responses of INC neurons **(A)** Current response of an INC neuron to local application of 1 mM acetylcholine (ACh). **(B)** Comparisons of the amplitude (1) and charge transfer (2) of the ACh-induced current between INC and PHN neurons. **(C,D)** Comparisons of the amplitude (1) and charge transfer (2) of the ACh-induced currents in glutamatergic **(C)** and inhibitory **(D)** neurons between the INC and the PHN. Plots indicate data obtained from individual neurons. The numbers in parentheses below each group label in B–D indicate the number of analyzed neurons. Asterisks indicate a significant difference between groups (**p < 0.01). Error bars represent the SDs.

We next investigated the proportion of mAChR- and nAChR-mediated currents in ACh-induced currents by the application of 5 μM atropine as an mAChR antagonist and 10 μM mecamylamine as an nAChR antagonist (Figures 2A, D). The analyses were performed using data that were successfully obtained from 16 glutamatergic and 16 inhibitory neurons in the INC, as well as 14 glutamatergic and 15 inhibitory PHN neurons previously reported in Zhang et al. (2016). The percentages of nAChR-mediated current (59.3 ± 20.0%) and charge (56.9 ± 23.3%) were not significantly different from the percentages of mAChR-mediated current (40.7 ± 20.0%, *p* = 0.084) and charge (43.1 ± 23.3%, *p* = 0.257) in glutamatergic INC neurons (Figure 2B), although the percentages of nAChR-mediated current (63.4 ± 13.2%) and charge (64.0 ± 11.7%) were significantly greater than the percentages of mAChR-mediated current (36.6 ± 13.2%, *p* = 0.0022) and charge (36.0 ± 11.7%, *p* = 0.0006) in glutamatergic PHN neurons (Figure 2E). In inhibitory neurons (Figures 2C, F), the percentages of nAChR-mediated current (INC: 62.0 ± 17.1%, PHN: 70.6 ± 18.8%) and charge (INC: 62.0 ± 22.0%, PHN: 66.9 ± 12.2%) were significantly greater than the percentages of mAChR-mediated current (INC: 39.0 ± 17.1%, *p* = 0.0212, PHN: 29.4 ± 18.8%, *p* = 0.0008) and charge (INC: 38.0 ± 22.0%, *p* = 0.0456, PHN: 33.1 ± 12.2, *p* = 0.0001).

**FIGURE 2:**
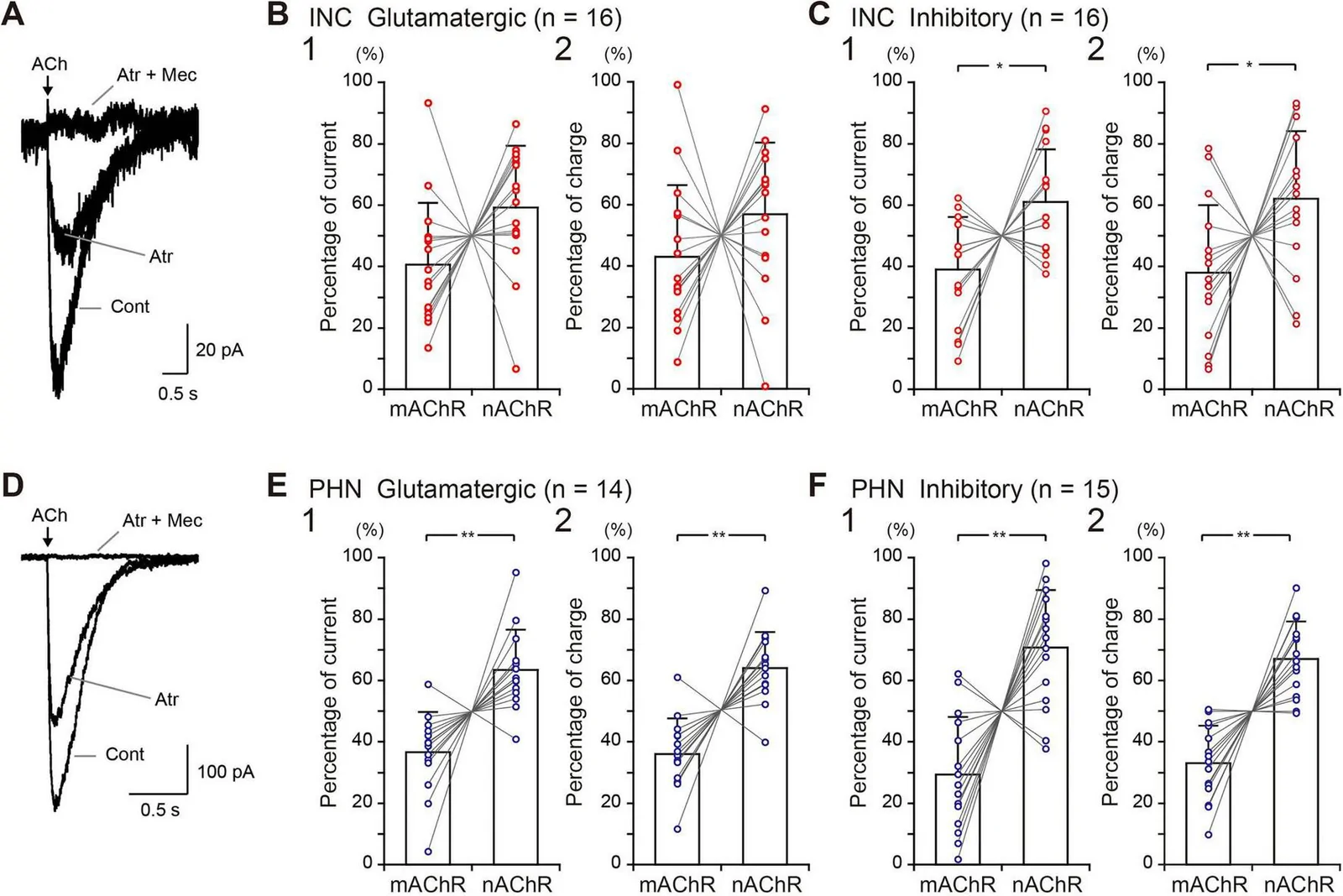
Comparison of current responses mediated by muscarinic and nicotinic acetylcholine receptors. **(A,D)** Representative current responses of an INC glutamatergic neuron **(A)** and a PHN glutamatergic neuron **(D)** to local application of 1 mM ACh. In both neurons, the ACh-induced currents were reduced by a muscarinic receptor antagonist, atropine (Atr, 5 μM), and a nicotinic receptor antagonist, mecamylamine (Mec, 10 μM). **(B,C,E,F)** Comparisons of the percentages of the amplitude and charge transfer between current responses mediated by muscarinic ACh receptors (mAChR) and those mediated by nicotinic ACh receptors (nAChR) in glutamatergic and inhibitory neurons in the INC **(B,C)** and the PHN **(E,F)** . Asterisks indicate a significant difference between groups (**p < 0.01, *p < 0.05). The number of analyzed neurons is indicated in the panel titles **(B,C,E,F)** .

### Primary origin of cholinergic inputs to the INC

In the INC of ChAT-tdTomato rats, tdTomato-expressing axonal fibers were observed (Figure 3A). To determine the brain regions that provide these cholinergic inputs, we unilaterally injected a retrograde AAV vector (AAVrg-hSyn-EGFP) into the INC (Figures 3B, C, *n* = 3). Although a few EGFP single-positive neurons were observed in the PPTg (Figures 3D1, D2) and the LDTg (Figures 3E1, E2), no EGFP/tdTomato double-labeled neurons (retrogradely labeled cholinergic neurons) were identified in these regions. In contrast, double-labeled neurons (ipsilateral: 14 ± 4 neurons per 3 sections; contralateral: 15 ± 3 neurons per 3 sections) were localized in the caudal region of the medial vestibular nucleus (MVN), particularly within its magnocellular region (MVeMC) (Paxinos and Watson, 2018), with the highest density observed between bregma −12.24 and −12.60 mm (Figures 3F1, F2). Although EGFP single-positive neurons were observed in the MVeMC (Figures 3F, G), the parvocellular region of the MVN (MVePC) (Figure 3G), and the PHN (data not shown), no double-labeled neurons were found in the MVePC or the PHN (Figures 3F, G). These results indicate that the INC receives cholinergic projections primarily from the caudal MVeMC.

**FIGURE 3:**
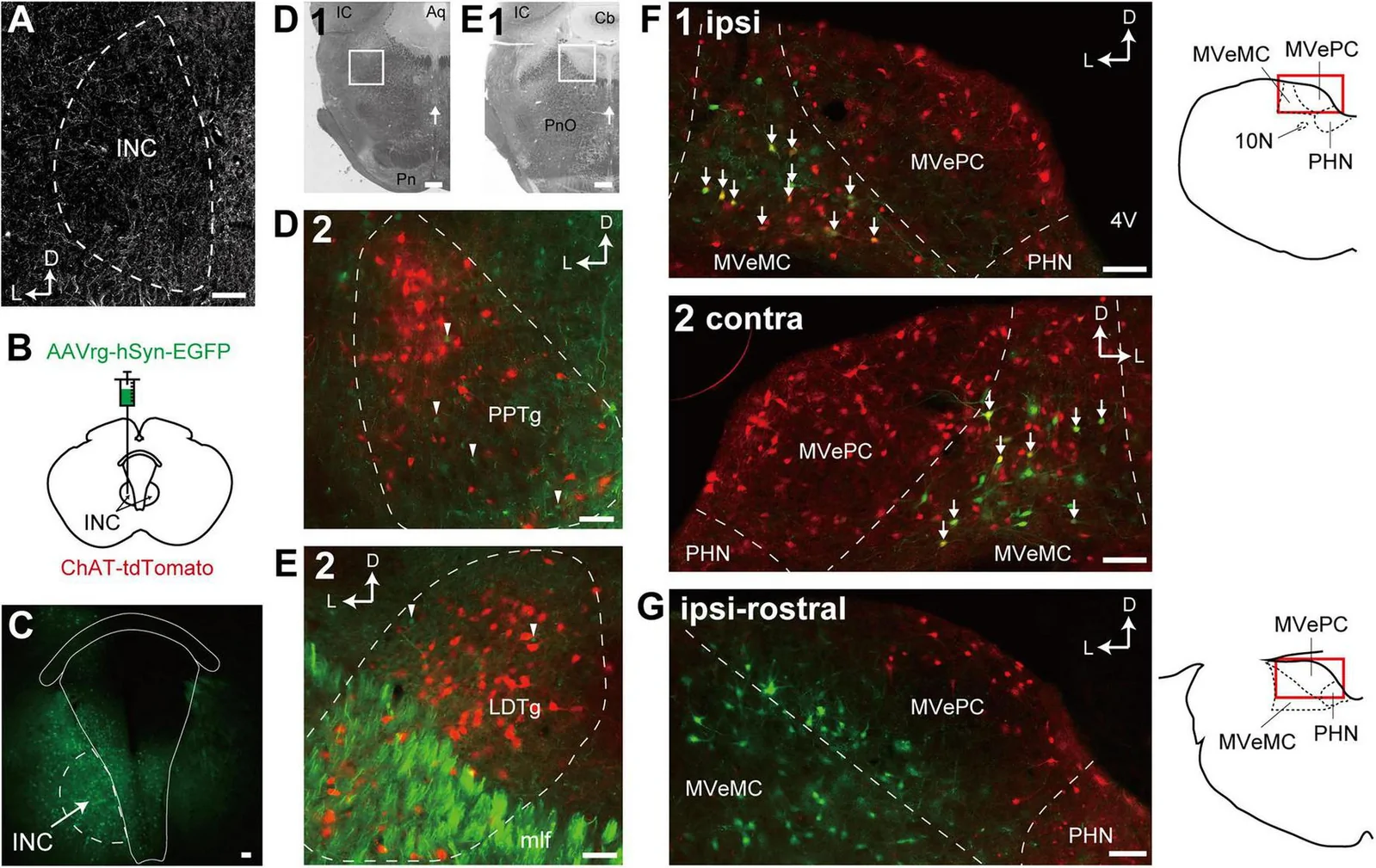
Cholinergic inputs to the INC originate primarily from the caudal MVN. **(A)** tdTomato-expressing axonal fibers in the INC of a ChAT-tdTomato rat. **(B)** Schematic drawing of the injection of AAVrg-hSyn-EGFP into the INC. **(C)** Fluorescence photomicrograph of the injection site. (D–G) Photomicrographs of frontal sections, including the ipsilateral pedunculopontine tegmental nucleus (PPTg) under bright-field (D1) and fluorescence (D2) microscopy, the ipsilateral laterodorsal tegmental nucleus (LDTg) under bright-field (E1) and fluorescence (E2) microscopy, the ipsilateral caudal region of the medial vestibular nucleus (MVN) and prepositus hypoglossi nucleus (PHN) (F1), the contralateral caudal region of the MVN and PHN (F2), and the ipsilateral rostral region of the PHN and MVN **(G)** . The white boxes in panels (D1) and (E1) indicate the areas shown in panels (D2) and (E2), respectively. The red bounding boxes in the semischematic atlas drawings next to panels (F1) and **(G)** indicate the imaging fields of panels (F1) and **(G)**, respectively. The arrowheads in panels (D2) and (E2) indicate representative EGFP single-positive neurons. The EGFP-positive fibers observed in the mlf (E2) likely represent the descending axons of INC neurons infected with the AAV vector at the injection site. The arrows in panels (D1) and (E1) indicate the midline. The arrows in panels (F1) and (F2) indicate neurons that express both EGFP and tdTomato. The dashed lines approximately indicate the anatomical boundaries of each nucleus. Scale bars: 500 μm in panels (D1) and (E1); 100 μm in all others. 10N, dorsal motor nucleus of the vagus nerve; 4V, fourth ventricle; Aq, cerebral aqueduct; Cb, cerebellum; IC, inferior colliculus; mlf, medial longitudinal fasciculus; MVeMC, magnocellular region of the MVN; MVePC, parvocellular region of the MVN; Pn, pontine nuclei; PnO, oral pontine reticular nucleus.

To compare the origins of cholinergic inputs between the vertical and horizontal neural integrators, we performed unilateral AAVrg injections into the PHN, with the viral spread extending to the adjacent MVN (Figures 4A1, A2; *n* = 3). Although the mesopontine tegmentum (PPTg and LDTg) has previously been suggested as a source of cholinergic inputs to the PHN, we observed no EGFP/tdTomato double-labeled neurons in either the PPTg (Figure 4B) or the LDTg (Figure 4C), despite the presence of a few EGFP single-positive neurons in these regions. To rule out the possibility of direct infection caused by viral spread on the injected side, we analyzed the MVN and PHN on the contralateral side. Double-labeled neurons (contralateral MVN: 6 ± 3 neurons per 3 sections; contralateral PHN: 2 ± 2 neurons per 3 sections) were observed primarily in the caudal region of the MVN, corresponding to the MVeMC, and the caudal PHN itself, with the highest density observed approximately between bregma −11.64 and −12.24 mm (Figure 4D). Furthermore, the successful retrograde transport of the AAV vector in this experiment was confirmed by the presence of retrogradely labeled neurons in both the INC (Figure 4E; Kokkoroyannis et al., 1996; Sugimura et al., 2024) and the oral pontine reticular nucleus (PnO) (Figure 4F; McCrea and Baker, 1985; Iwasaki et al., 1999), which are known to provide inputs to the PHN.

**FIGURE 4:**
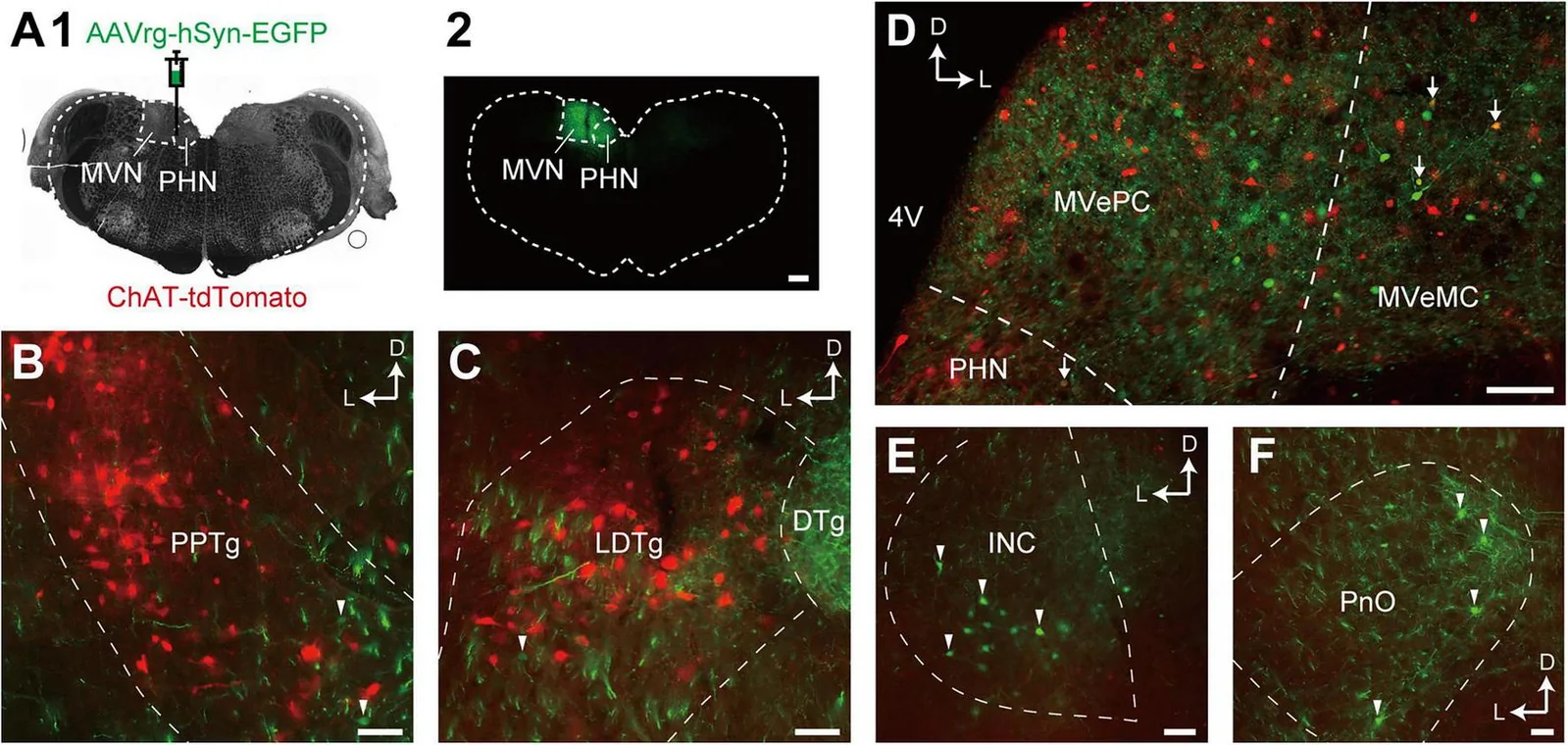
Cholinergic inputs to the PHN originate primarily from the caudal MVN and the PHN itself. **(A1)** Schematic drawing of the injection of AAVrg-hSyn-EGFP into the PHN of ChAT-tdTomato rats superimposed on a bright-field photomicrograph of a frontal brainstem section. **(A2)** Fluorescence photomicrograph of the injection site. **(B–F)** Fluorescence photomicrographs of frontal sections following PHN injection, including the ipsilateral pedunculopontine tegmental nucleus (PPTg) **(B)**, the ipsilateral laterodorsal tegmental nucleus (LDTg) **(C)**, the contralateral PHN and medial vestibular nucleus (MVN) **(D)**, the ipsilateral interstitial nucleus of Cajal (INC) **(E)**, and the ipsilateral oral pontine reticular nucleus (PnO) **(F)** . The arrowheads in panels **(B,C,E,F)** indicate representative EGFP single-positive neurons. The arrows in panel **(D)** indicate neurons that express both EGFP and tdTomato. The dashed lines approximately indicate the anatomical boundaries of each nucleus. The approximate location of the PnO is indicated in the low-magnification image shown in Figure 3E1. Scale bars: 500 μm in panel **(A2)**; 100 μm in all others. 4V, fourth ventricle; DTg, dorsal tegmental nucleus; MVeMC, magnocellular region of the MVN; MVePC, parvocellular region of the MVN.

These anatomical findings reveal that the primary source of cholinergic inputs to the INC is the caudal MVN rather than the mesopontine tegmentum. Similarly, cholinergic inputs to the PHN also originate from lower brainstem structures, including the caudal MVN and the PHN itself.

## Discussion

In the present study, we demonstrated that local application of ACh induces inward currents in INC neurons, providing evidence that cholinergic inputs produce an excitatory effect on the neural integrator for vertical gaze holding. Pharmacological analyses revealed that both nAChRs and mAChRs are involved in these responses, exhibiting a cell type-specific receptor dependency distinct from that of the PHN. Furthermore, retrograde tracing demonstrated that the primary origin of cholinergic inputs to the INC is the caudal MVN, whereas the PHN receives inputs from both the caudal MVN and the PHN itself. From these findings, we obtained answers to the three questions raised in the Introduction: (1) INC neurons exhibit excitatory inward currents in response to ACh; (2) these responses involve both nAChRs and mAChRs, which exhibit cell type-specific dependency; and (3) the primary origin of cholinergic inputs to the INC is the caudal MVN, whereas the PHN additionally receives inputs from the caudal PHN itself. These answers indicate that the INC is modulated by cholinergic transmission originating primarily from the caudal MVN.

With respect to the mechanisms underlying this excitatory cholinergic modulation, previous studies have shown that mAChRs play a primary role in the horizontal neural integrator (PHN) (Navarro-López et al., 2004; Delgado-García et al., 2006). Activation of mAChRs provides a continuous depolarizing drive to PHN neurons. This drive enables these neurons to sustain their firing after receiving transient burst inputs (velocity signals) from premotor burst neurons. Based on our finding that mAChRs mediate inward currents in INC neurons, a similar mAChR-dependent depolarizing drive likely enables INC neurons to sustain their firing. Whereas previous models for horizontal integrators have focused primarily on mAChRs, we have previously shown that nAChRs also significantly contribute to driving repetitive firing in PHN neurons (Zhang et al., 2016, 2018). Consistent with these previous findings, the results of the present study revealed that nAChRs, as well as mAChRs, are involved in the ACh responses of INC neurons. Thus, rather than functioning through mAChRs alone, the combined activation of both nAChRs and mAChRs may contribute to the sustained neuronal activity required for vertical gaze holding. Although ACh-induced inward currents were observed in INC neurons, the present study did not examine whether these responses enhance the firing rate or persistent spiking. Thus, the present findings demonstrate cholinergic modulation at the membrane current level but do not directly establish a link between cholinergic signaling and gaze holding.

Although both neural integrators receive excitatory cholinergic modulation, they exhibit differences in their response properties to ACh. We observed that the amplitude and charge transfer of the ACh-induced currents in INC neurons were smaller than those in PHN neurons, which may reflect a lower density of ACh receptors in INC neurons. In both nuclei, ACh induced inward currents in both glutamatergic and inhibitory neurons, indicating that cholinergic inputs activate both excitatory and inhibitory elements to modulate the overall dynamics of the local network. However, in the INC, the relative contributions of nAChRs and mAChRs to these currents differed depending on the cell type; the contribution of nAChRs was significantly greater than that of mAChRs in inhibitory neurons, whereas their contributions were not significantly different in glutamatergic neurons. In contrast, nAChRs consistently predominated in both cell types in the PHN (Figures 2E, F). This target–nucleus-specific difference in cholinergic modulation follows a similar pattern to our previous finding that the INC and PHN utilize distinct receptor mechanisms (predominantly NMDA- and Ca^2+^-permeable AMPA receptors, respectively) within their local excitatory networks (Saito and Yanagawa, 2010; Saito et al., 2017; Saito and Sugimura, 2020), indicating that both integrators exhibit distinct receptor dependencies for not only their base circuits but also their modulatory systems.

Our retrograde tracing results demonstrated that the primary origin of cholinergic inputs to the INC is the MVN. These cholinergic projection neurons were localized to the caudal region, corresponding to the MVeMC, and no such neurons were found in the rostral region. The MVN processes vestibular signals, and its rostral and caudal regions are known to play distinct functional roles based on their input-output connections (Leigh and Zee, 2015). The rostral MVN receives primary vestibular afferents and projects directly to the ocular motor nuclei, thereby mediating the vestibulo-ocular reflex (McCrea et al., 1987; Barmack, 2003; Leigh and Zee, 2015). In contrast, the caudal MVN, particularly the MVeMC, is reciprocally connected with the vestibulocerebellum (Barmack, 2003; Leigh and Zee, 2015), which is crucial for optimizing gaze stabilization (Waespe et al., 1985; Wearne et al., 1998). Because cholinergic mechanisms underlie the persistent neural activity necessary for gaze holding (Navarro-López et al., 2004; Delgado-García et al., 2006), this specific cholinergic input from the MVeMC may drive the sustained firing of the neural integrators based on vestibular information associated with this cerebellar loop. Furthermore, our study demonstrated that the primary origin of cholinergic inputs to the PHN is the caudal MVN and the PHN itself. These results contrast with previous assumptions that the mesopontine tegmentum is the primary cholinergic source for the PHN (Navarro-López et al., 2004; Delgado-García et al., 2006). However, although previous anatomical studies have shown connections between the mesopontine tegmentum and the PHN (Higo et al., 1990; Iwasaki et al., 1999), there are no direct data demonstrating that these projections are cholinergic (Higo et al., 1990). The mesopontine tegmentum is known to be involved in state-dependent arousal, sleep-wake cycles, and attentional processes (Steriade et al., 1990; Oken et al., 2006; Brown et al., 2012; Scammell et al., 2017). Because our focal viral injections did not cover the entire PHN, we do not necessarily rule out the possibility of such arousal-related cholinergic inputs from the mesopontine tegmentum. Rather, our finding that the caudal MVN serves as a cholinergic source suggests that cholinergic modulation of the PHN conveys signals closely coupled with vestibular information. The shared cholinergic projections from the MVeMC to the vertical and horizontal neural integrators provide an anatomical and physiological basis for coordinating three-dimensional gaze holding.

With respect to the methodological considerations of anatomical tracing, we initially tested a fluorescent dextran tracer used in our previous studies (e.g., Sugimura et al., 2025). However, reliable identification of retrogradely labeled somata was technically challenging because their punctate fluorescent signals were difficult to distinguish from those of fluorescently labeled fibers originating from the INC and passing through the mlf. We therefore utilized AAVrg-EGFP, which provides diffuse cytoplasmic fluorescence and facilitates clear identification of retrogradely labeled neurons. Although potential bias due to viral tropism cannot be entirely ruled out, the successful labeling of cholinergic neurons in the caudal MVeMC suggests that the absence of labeled cholinergic neurons in the LDTg and PPTg is unlikely to be explained solely by this factor. Although viral injections centered on the INC exhibited limited spread into adjacent mesencephalic structures on the injected side, this localized spread is unlikely to affect our conclusions. The fluorescence observed on the contralateral side reflects EGFP-labeled axons crossing the posterior commissure (Leigh and Zee, 2015) rather than direct viral diffusion. Moreover, despite the local spread around the INC, no double-labeled cholinergic neurons were detected in the LDTg or PPTg. These findings suggest that neither the INC nor adjacent midbrain regions receive substantial cholinergic input from these mesopontine nuclei. Instead, the robust labeling observed in the caudal MVeMC supports its role as the principal cholinergic source for the vertical neural integrator.

It is important to acknowledge the methodological limitations of the pharmacological approach used in the present study. We utilized localized puff application of 1 mM ACh to investigate cholinergic responses. However, the applied ACh is substantially diluted before it reaches the recorded neuron. This localized application does not replicate the complex temporal and spatial dynamics of endogenous cholinergic transmission. Instead, the purpose of this protocol was to evaluate receptor-mediated response capability under standardized and saturating conditions by positioning the puff pipette at a distance where ACh-induced responses reached saturation. Because somatic voltage-clamp recordings are subject to space-clamp limitations in distal dendritic compartments, the absolute conductance at remote dendritic sites may be underestimated. However, our local application protocol, in which the puff pipette was positioned near the cell soma as previously described (Zhang et al., 2014, 2018), is expected to restrict ACh action predominantly at the soma and proximal dendrites, unlike bath application. Since these proximal regions are more effectively voltage clamped than distal dendritic compartments are, the impact of space-clamp artifacts on the relative comparisons of current amplitudes and charge transfer between the INC and PHN groups is likely reduced. Nevertheless, this methodological limitation should be considered when interpreting the absolute magnitude of ACh-induced responses.

More broadly, the present findings should also be interpreted within the limitations of slice preparation. While the present slice recordings characterize the intrinsic cholinergic receptor properties of INC neurons, the actual amplitudes of ACh-induced currents and the relative contributions of nAChR- and mAChR-mediated components under *in vivo* conditions, where background synaptic inputs and neuromodulatory influences are present, should be interpreted with caution. Oculomotor neural integrators are regulated by complex interactions between fast synaptic transmission (glutamatergic and GABAergic) and multiple neuromodulatory pathways, including cholinergic, noradrenergic, serotonergic, and purinergic pathways (Saito and Yanagawa, 2010; Shino et al., 2011; Sugioka and Saito, 2018; Saito and Sugimura, 2020, 2023, 2024, 2025). Although the present study focused on cholinergic regulation rather than the integrated actions of multiple signaling pathways operating in vivo, our findings clarify the intrinsic cholinergic properties of neural integrator neurons, providing an important foundation for understanding these complex neuromodulatory interactions.

In conclusion, the present electrophysiological and anatomical data demonstrate that both vertical and horizontal neural integrators receive cholinergic modulation originating from the caudal MVN. However, to process these signals, they utilize distinct internal mechanisms, such as cell type-specific receptor dependencies. Thus, common modulatory sources from the caudal MVN may modulate intrinsic networks for each integrator involved in three-dimensional gaze holding.

## Data Availability

The raw data supporting the conclusions of this article will be made available by the authors, without undue reservation.
